# Supplementary material for: COZOID: contact zone identifier for visual analysis of protein-protein interactions
Source: BMC Bioinformatics. 2018 Apr 6;19:125. doi: 10.1186/s12859-018-2113-6 (PMC5889581; doi:10.1186/s12859-018-2113-6)
Supplement: Supplementary file 2 — Software build. Executable binary file of the software tool. (ZIP 118,784 kb) [file 12859_2018_2113_MOESM2_ESM.zip › cozoid/java/docs/beansbinding-1.2.1-doc.zip!/org/jdesktop/beansbinding/BeanProperty.html]

BeanProperty (Beans Binding)


---


|  |  |  |
| --- | --- | --- |
| **Overview** \| **Package** \| **Class** \| **Use** \| **Tree** \| **Deprecated** \| **Index** \| **Help** | |  |
| **PREV CLASS**   **NEXT CLASS** | **FRAMES**    **NO FRAMES**     **All Classes** |  |
| SUMMARY: NESTED \| FIELD \| CONSTR \| METHOD | DETAIL: FIELD \| CONSTR \| METHOD |  |


---


## org.jdesktop.beansbinding Class BeanProperty<S,V>

```
java.lang.Object
  org.jdesktop.beansbinding.Property<S,V>
      org.jdesktop.beansbinding.PropertyHelper<S,V>
          org.jdesktop.beansbinding.BeanProperty<S,V>
```

**Type Parameters:**: `S` - the type of source object that this `BeanProperty` operates on: `V` - the type of value that this `BeanProperty` represents

---

``` public final class BeanProperty<S,V> extends PropertyHelper<S,V> ```

An implementation of `Property` that uses a simple dot-separated path
syntax to address Java Beans properties of source objects. For example, to
create a property representing the `firstName` property of an obect:

```
    BeanProperty.create("firstName");
```

Or to create a property representing the `firstName` property of
an object's `mother` property:

```
    BeanProperty.create("mother.firstName");
```

An instance of `BeanProperty` is immutable and can be used with
different source objects. When a `PropertyStateListener` is added to
a `BeanProperty` for a given source object, the `BeanProperty`
starts listening to all objects along the path (based on that source object)
for change notification, and reflects any changes by notifying the
listener associated with the property for that source object. So, in the second
example above, if a `PropertyStateListener` is added to the property
for an object `Duke`, the `PropertyStateListener` is notified
when either `Duke's` mother changes (if the new mother's name is
different), or `Duke's mother's firstName` changes.

It is very important that any bean properties addressed via a `BeanProperty`
follow the Java Beans specification, including firing property change notification;
otherwise, `BeanProperty` cannot respond to change. As some beans outside
of your control may not follow the Java Beans specification, `BeanProperty`
always checks the `BeanAdapterFactory` to
see if a delegate provider has been registered to provide a delegate bean to take
the place of an object for a given property. See the
ext package level documentation for more
details.

When there are no `PropertyStateListeners` installed on a `BeanProperty`
for a given source, all `Property` methods act by traversing the entire
path from the source to the end point, thereby always providing "live" information.
On the contrary, when there are `PropertyStateListeners` installed, the beans
along the path (including the final value) are cached, and only updated upon
notification of change from a bean. Again, this makes it very important that any
bean property that could change along the path fires property change notification.

**Readability** of a `BeanProperty` for a given source is defined as follows:
*A `BeanProperty` is readable for a given source if and only if
a) each bean in the path, starting with the source, defines a Java Beans getter
method for the the property to be read on it AND b) each bean in the path,
starting with the source and ending with the bean on which we read the final
property, is `non-null`. The final value being `null` does not
affect the readability.*

So, in the second example given earlier, the `BeanProperty` is readable for (@code Duke} when all
of the following are true: `Duke` defines a Java Beans getter for
`mother`, `Duke's mother` defines a Java Beans getter for
`firstName`, `Duke` is `non-null`, `Duke's mother`
is `non-null`. The `BeanProperty` is therefore unreadable when
any of the following is true: `Duke` does not define a Java Beans
getter for `mother`, `Duke's mother` does not define a Java
Beans getter for `firstName`, `Duke` is `null`,
`Duke's mother` is `null`.

**Writeability** of a `BeanProperty` for a given source is defined as follows:
*A `BeanProperty` is writeable for a given source if and only if
a) each bean in the path, starting with the source and ending with the bean on
which we set the final property, defines a Java Beans getter method for the
property to be read on it AND b) the bean on which we set the final property
defines a Java Beans setter for the property to be set on it AND c) each bean
in the path, starting with the source and ending with the bean on which we
set the final property, is `non-null`. The final value being `null`
does not affect the writeability.*

So, in the second example given earlier, the `BeanProperty` is writeable for `Duke` when all
of the following are true: `Duke` defines a Java Beans getter for
`mother`, `Duke's mother` defines a Java Beans setter for
`firstName`, `Duke` is `non-null`, `Duke's mother`
is `non-null`. The `BeanProperty` is therefore unreadable when
any of the following is true: `Duke` does not define a Java Beans
getter for `mother`, `Duke's mother` does not define a Java
Beans setter for `firstName`, `Duke` is `null`,
`Duke's mother` is `null`.

In addition to working on Java Beans properties, any object in the path
can be an instance of `Map`. In this case, the `Map's get`
method is used with the property name as the getter, and the
`Map's put` method is used with the property name as the setter.
`BeanProperty` can only respond to changes in `Maps`
if they are instances of `ObservableMap`.

Some methods in this class document that they can throw
`PropertyResolutionException` if an exception occurs while trying
to resolve the path. The throwing of this exception represents an abnormal
condition and if listeners are installed for the given source object,
leaves the `BeanProperty` in an inconsistent state for that source object.
A `BeanProperty` should not be used again for that same source object
after such an exception without first removing all listeners associated with
the `BeanProperty` for that source object.

---

| **Method Summary** | |
| --- | --- |
| `static <S,V> BeanProperty<S,V>` | `create(Property<S,?> baseProperty, java.lang.String path)`             Creates an instance of `BeanProperty` for the given base property and path. |
| `static <S,V> BeanProperty<S,V>` | `create(java.lang.String path)`             Creates an instance of `BeanProperty` for the given path. |
| `V` | `getValue(S source)`             Returns the value of this `Property` for the given source. |
| `java.lang.Class<? extends V>` | `getWriteType(S source)`             Returns the type of object that is suitable for setting as the value of this `Property` by calls to `setValue`. |
| `boolean` | `isReadable(S source)`             Returns whether or not the `Property` is readable for the given source. |
| `boolean` | `isWriteable(S source)`             Returns whether or not the `Property` is writeable for the given source. |
| `protected  void` | `listeningStarted(S source)`             Called when this `PropertyHelper` changes from having no listeners installed for the given source object to having listeners installed for the given source object. |
| `protected  void` | `listeningStopped(S source)`             Called when this `PropertyHelper` changes from having listeners installed for the given source object to having no listeners installed for the given source object. |
| `void` | `setValue(S source, V value)`             Sets the value of this `Property` for the given source. |
| `java.lang.String` | `toString()`             Returns a string representation of the `BeanProperty`. |

**Methods inherited from class org.jdesktop.beansbinding.PropertyHelper**

`addPropertyStateListener, firePropertyStateChange, getPropertyStateListeners, isListening, removePropertyStateListener`

**Methods inherited from class java.lang.Object**

`clone, equals, finalize, getClass, hashCode, notify, notifyAll, wait, wait, wait`

**Method Detail**

### create

```
public static final <S,V> BeanProperty<S,V> create(java.lang.String path)
```

:   Creates an instance of `BeanProperty` for the given path.

    :   **Parameters:**: `path` - the path **Returns:**: an instance of `BeanProperty` for the given path **Throws:**: `java.lang.IllegalArgumentException` - if the path is null, or contains no property names

---


### create

```
public static final <S,V> BeanProperty<S,V> create(Property<S,?> baseProperty,
                                                   java.lang.String path)
```

:   Creates an instance of `BeanProperty` for the given base property
    and path. The path is relative to the value of the base property.

    :   **Parameters:**: `baseProperty` - the base property: `path` - the path **Returns:**: an instance of `BeanProperty` for the given base property and path **Throws:**: `java.lang.IllegalArgumentException` - if the path is null, or contains no property names

---


### getWriteType

```
public java.lang.Class<? extends V> getWriteType(S source)
```

:   Returns the type of object that is suitable for setting as the value
    of this `Property` by calls to `setValue`.

    See the class level documentation for the definition of writeability.

    :   **Specified by:**: `getWriteType` in class `PropertyHelper<S,V>`
    :   **Parameters:**: `source` - the source object on which to operate **Returns:**: the type of object suitable for setting as the value **Throws:**: `java.lang.UnsupportedOperationException` - if the `Property` is not writeable for the given source: `PropertyResolutionException` - if an exception occurs while resolving the path **See Also:**: `setValue(S, V)`, `isWriteable(S)`

---


### getValue

```
public V getValue(S source)
```

:   Returns the value of this `Property` for the given source.

    See the class level documentation for the definition of readability.

    :   **Specified by:**: `getValue` in class `PropertyHelper<S,V>`
    :   **Parameters:**: `source` - the source object on which to operate **Returns:**: the value of this `Property` for the given source **Throws:**: `java.lang.UnsupportedOperationException` - if the `Property` is not readable for the given source: `PropertyResolutionException` - if an exception occurs while resolving the path **See Also:**: `isReadable(S)`

---


### setValue

```
public void setValue(S source,
                     V value)
```

:   Sets the value of this `Property` for the given source.

    See the class level documentation for the definition of writeability.

    :   **Specified by:**: `setValue` in class `PropertyHelper<S,V>`
    :   **Parameters:**: `source` - the source object on which to operate: `value` - the new value for the `Property` **Throws:**: `java.lang.UnsupportedOperationException` - if the `Property` is not writeable for the given source: `PropertyResolutionException` - if an exception occurs while resolving the path **See Also:**: `isWriteable(S)`, `getWriteType(S)`

---


### isReadable

```
public boolean isReadable(S source)
```

:   Returns whether or not the `Property` is readable for the given source.

    See the class level documentation for the definition of readability.

    :   **Specified by:**: `isReadable` in class `PropertyHelper<S,V>`
    :   **Parameters:**: `source` - the source object on which to operate **Returns:**: whether or not the `Property` is readable for the given source. **Throws:**: `java.lang.UnsupportedOperationException`: `PropertyResolutionException` - if an exception occurs while resolving the path **See Also:**: `isWriteable(S)`

---


### isWriteable

```
public boolean isWriteable(S source)
```

:   Returns whether or not the `Property` is writeable for the given source.

    See the class level documentation for the definition of writeability.

    :   **Specified by:**: `isWriteable` in class `PropertyHelper<S,V>`
    :   **Parameters:**: `source` - the source object on which to operate **Returns:**: whether or not the `Property` is writeable for the given source. **Throws:**: `java.lang.UnsupportedOperationException`: `PropertyResolutionException` - if an exception occurs while resolving the path **See Also:**: `isReadable(S)`

---


### listeningStarted

```
protected final void listeningStarted(S source)
```

:   **Description copied from class: `PropertyHelper`**
:   Called when this `PropertyHelper` changes from having
    no listeners installed for the given source object to
    having listeners installed for the given source object. This
    is the ideal time for subclasses to install any listeners needed
    to track change on the source object.

    :   **Overrides:**: `listeningStarted` in class `PropertyHelper<S,V>`
    :   **See Also:**: `PropertyHelper.listeningStopped(S)`

---


### listeningStopped

```
protected final void listeningStopped(S source)
```

:   **Description copied from class: `PropertyHelper`**
:   Called when this `PropertyHelper` changes from having
    listeners installed for the given source object to
    having no listeners installed for the given source object. This
    is the ideal time for subclasses to remove any listeners that
    they've installed to track changes on the source object.

    :   **Overrides:**: `listeningStopped` in class `PropertyHelper<S,V>`
    :   **See Also:**: `PropertyHelper.listeningStopped(S)`

---


### toString

```
public java.lang.String toString()
```

:   Returns a string representation of the `BeanProperty`. This
    method is intended to be used for debugging purposes only, and
    the content and format of the returned string may vary between
    implementations. The returned string may be empty but may not
    be `null`.

    :   **Overrides:**: `toString` in class `java.lang.Object`
    :   **Returns:**: a string representation of this `BeanProperty`


---


|  |  |  |
| --- | --- | --- |
| **Overview** \| **Package** \| **Class** \| **Use** \| **Tree** \| **Deprecated** \| **Index** \| **Help** | |  |
| **PREV CLASS**   **NEXT CLASS** | **FRAMES**    **NO FRAMES**     **All Classes** |  |
| SUMMARY: NESTED \| FIELD \| CONSTR \| METHOD | DETAIL: FIELD \| CONSTR \| METHOD |  |


---
